# Intuitive Web-Based Experimental Design for High-Throughput Biomedical Data

**DOI:** 10.1155/2015/958302

**Published:** 2015-04-14

**Authors:** Andreas Friedrich, Erhan Kenar, Oliver Kohlbacher, Sven Nahnsen

**Affiliations:** ^1^Applied Bioinformatics, Center for Bioinformatics, Quantitative Biology Center (QBiC) and Department of Computer Science, University of Tübingen, 72076 Tübingen, Germany; ^2^Quantitative Biology Center (QBiC), University of Tübingen, 72076 Tübingen, Germany

## Abstract

Big data bioinformatics aims at drawing biological conclusions from huge and complex biological datasets. Added value from the analysis of big data, however, is only possible if the data is accompanied by accurate metadata annotation. Particularly in high-throughput experiments intelligent approaches are needed to keep track of the experimental design, including the conditions that are studied as well as information that might be interesting for failure analysis or further experiments in the future. In addition to the management of this information, means for an integrated design and interfaces for structured data annotation are urgently needed by researchers. Here, we propose a factor-based experimental design approach that enables scientists to easily create large-scale experiments with the help of a web-based system. We present a novel implementation of a web-based interface allowing the collection of arbitrary metadata. To exchange and edit information we provide a spreadsheet-based, humanly readable format. Subsequently, sample sheets with identifiers and metainformation for data generation facilities can be created. Data files created after measurement of the samples can be uploaded to a datastore, where they are automatically linked to the previously created experimental design model.

## 1. Introduction

Over the past years, the amount of data produced by the measurement of different parts of biological systems has become increasingly larger, with petabytes of data stored at repositories like the one found at the European Bioinformatics Institute (EBI), highlighting that biology has arrived in the big data environment [1]. Automation and more precise systems have led to the possibility to produce biomedical data in a high-throughput fashion. Perhaps the most famous example of this can be found in the field of genomics, which has been revolutionized by the development of next-generation sequencing (NGS) technologies that nearly replaced the Sanger sequencing approach [2]. With the rise of faster, cheaper, and more precise genomic sequencing the number of experiments and amount of data have exploded [3], a trend continuing with new third generation sequencing systems like the Illumina HiSeq X Ten [4]. Furthermore, many biomedical research projects are not limited to a single layer of genomics or proteomics data but aim instead at comprehensively profiling biological systems using the so-called multiomics approaches [5].

Big data is notable not only because of its size but also because of its relationality to other data and it has therefore been noted that one of the challenges connected to big data is descriptive [6]. Context is often required if data is to be analyzed or processed. In respect of life sciences this pertains to the experimental setup of a project. For most analyses done in high-throughput biology today, bioinformaticians or statisticians require metainformation about the data for its interpretation, especially when considering experiments comparing replicates of multiple different species, genotypes, or growth conditions for cell cultures.

Apart from providing the minimal information needed to analyse experimental data, these metadata are essential for almost anything that can be done with data beyond the initial experiment. It is especially important in view of sharing of high-throughput data [7–9]. The age of big data will provide an unprecedented opportunity for researchers to reuse data collected in previous experiments by themselves or others in the scientific community. Aims can range from the classic scientific approach of comparing new results to an older experiment to large-scale data mining approaches [10].

In recent years, community efforts have been undertaken to develop standards for data annotation and the sharing of metadata. An early example is MIAME, which describes the minimum information about a microarray experiment [11], and the microarray gene expression markup language MAGE-ML [12] aiming at annotating microarray experiments for sharing among researchers so they can be independently verified. While markup language-based formats are very powerful tools to store metadata relationships, their use is often impractical for laboratories in the life sciences lacking bioinformatics support. More commonly, spreadsheet files are used to share metadata, because they are humanly readable and easy to parse and to translate into databases and they can be edited by a wide range of software. One example of this is the MAGE-TAB format [13], created especially for ease of use in laboratories lacking computer science personnel.

The open Biological Experiment Browser (openBEB) is a framework for data acquisition and annotation in systems biology [14]. Metadata is also saved in an XML format, but the user is presented with a GUI to simplify input of new information. This is an interesting approach that can be extended to other technologies via plugins but requires the installation of software on the side of collaborating laboratories and its complexity especially targets biologists working closely with engineers and computer scientists.

In the life sciences, biological samples build the basis of any experiment. Biological experiments are commonly based on many samples to increase statistical power [15], where each sample is associated with distinct properties that describe it. An electronic capture of these parameters and a clear association with the subsequently acquired high-throughput data is a key requirement for automated data analysis and ultimately for the application of big data methods. However means and strategies for capturing and sharing these data remain elusive. With the advent of high-throughput, multicentered research, large-scale, data-driven experiments are frequently taking place in many different laboratories. Sample treatment in many different locations needs stringent modeling of multiple identifiers that are associated with the sample and its metadata, leading to the need for mapping or conversion steps.

It is often instrumental to coordinate this effort from a central place. In general, the facility where data and metadata are stored is a logical solution. This approach also provides an opportunity to store metadata before the data is created, which can often be a sensible choice. Having metadata and experimental design already stored provides means to check correctness of incoming data and can speed up processing.

Here, we propose a method to allow researchers to quickly design experiments containing a large number of samples. Our approach keeps track of both internal connections between involved organisms, tissue extracts, and prepared samples of experiments handled at multiple different centers as well as their metadata. We implemented a web-based wizard for maximum accessibility on different systems and designed a GUI.

Our approach not only allows collaborators to access their metadata or experimental design information via downloadable spreadsheets but also supports automated creation of parameter files for processing of the data in bioinformatics pipelines. Additionally, well-annotated data can facilitate reuse of data for future research, leading to experiments with larger correlative power.

## 2. Methods and Implementation

### 2.1. Metadata Management

**Figure 1:**
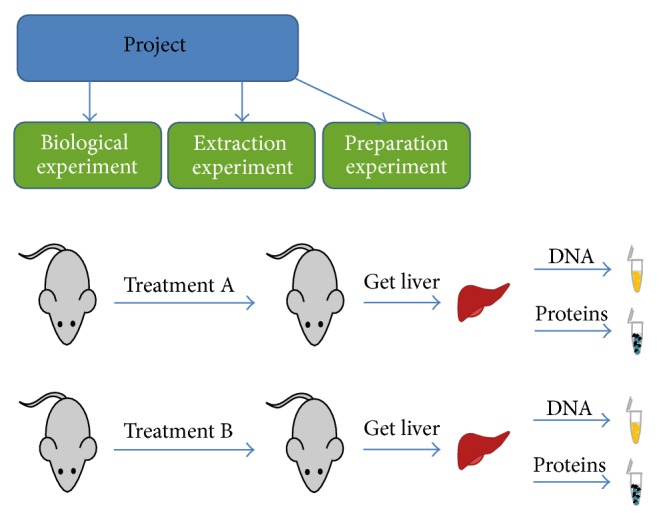
Data model used for experimental design: each project can contain multiple experiments of varying types: biological experiments contain biological entities like patients, animals, or plants used in the experiment and their metadata, for example, treatments or genotypes. Sample extraction experiments contain information about tissues or cells extracted from these entities and sample preparation experiments contain samples that have been prepared for data creation by mass spectrometry or other methods. Additionally, fields in the sample model allow for storage of common metadata like taxonomic identifiers, the type of measurement technology, instrument parameters, and so forth.

There are commonly multiple steps involved in the sample preparation of a biological experiment, each potentially being associated with its own set of metadata. In order to keep track of samples and data generated from them, the customized data model as visualized in Figure 1 represents a 3-tier experiment: the first step describes biological entities like patients, model organisms, or any other biological systems of interest. At this level the species can be selected from a predefined vocabulary taken from NCBI taxonomy [17] and other metainformation, such as drug treatments, genotypes, or phenotypes which can be added to the experiment and attached samples. The second step describes extraction of cells or tissues from the aforementioned organisms. Again, different metadata like growth conditions can be added at this level in addition to the specified tissues. The last step describes the preparation of samples for the actual data collection systems like next-generation sequencing or other methods. Here, information concerning library preparation is collected. Additionally, every sample type also allows the addition of specific information, for example, an internal identifier to connect samples to other databases, a secondary, humanly readable name, or other additional information concerning the sample. All these metadata and relations between samples can be stored in a relational database of choice. In addition, new models and steps, for example, for the collection of NGS- or mass spectrometry- (MS-) specific experimental metadata, can be easily added following the same principles.

To be able to uniquely match lab samples and created data to our virtual data model, we generate a unique identifier and barcodes that can be scanned using barcode readers. The identifier contains a weighted checksum digit and is used to name the data files created in experiments to simplify automated processing using Extract Transform Load (ETL) scripts to extract further metadata. The files are then moved to a connected datastore server, linking them to the data model in the process.

Since metadata in biological experiments can be highly variable, we provide the possibility to record a diverse set of information using a simple XML schema. This allows for both easy parsing and reusability of the data as well as an on-the-fly validation of the input data. Our schema can store both categorical metadata and continuous information, which enforces SI units.

### 2.2. Portlet

For our web application we created a Java portlet running on a Tomcat 7 server using Vaadin. Vaadin is an open source framework based on Ajax and Google Web Toolkit, meaning that most of the program logic takes place in the server-side, while the user is presented with an HTML5 and JavaScript interface [18].

**Figure 2:**
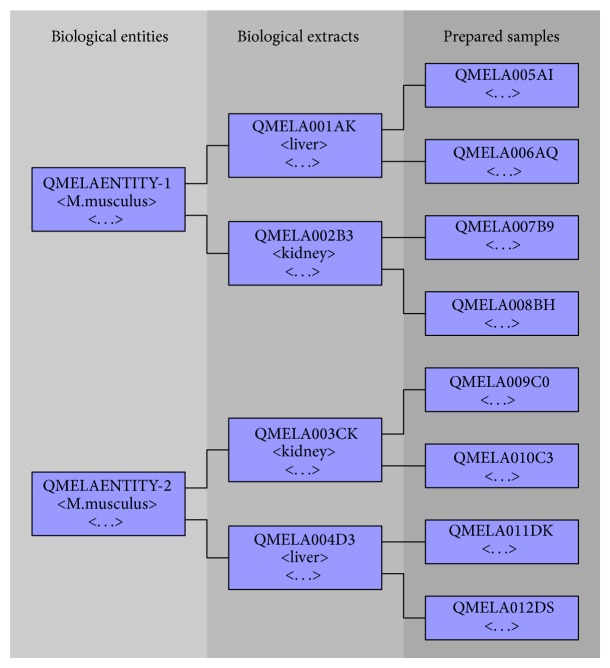
A simple visual representation of the three tiers (left to right) of an experimental design created by the portlet and their internal relations. With the exception of biological entities, each sample carries a project-specific identifier. Each tier contains unique metainformation like organism for entities or tissue for extracts. yEd graph editor (yFiles software) was employed for graphs visualization [16].

To create the instances tailored to our multistep experiment model and handle biological or technical replicates of each step, a factor-based experimental design approach was implemented, essentially building up the list of samples for each step by creating all permutations of user-specified conditions multiplied by replicates and attaching their hierarchical connections to each other (see Figure 2). Since this approach assumes a completely symmetric experimental design containing the same number of samples for each condition, the user can deselect superfluous samples after each step.

## 3. Results and Discussion

**Figure 3:**
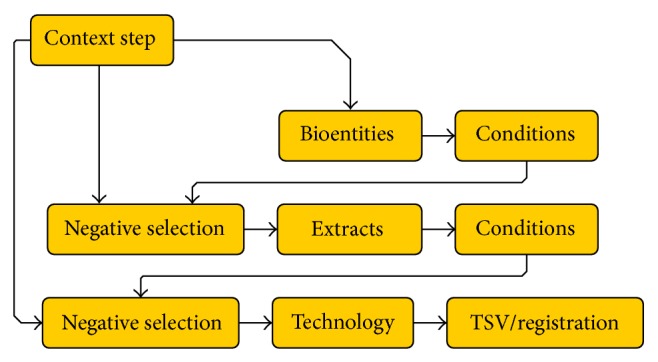
Simplified schema of the experimental design wizard's steps: In a first context step, users can choose to create a completely new experiment and to add a sample extraction experiment or just measurements to existing experiments in the system. Depending on context, sample data is either taken from the system or newly created from user input concerning the amount of replicates, species, and other metadata. After each step, samples can be deselected in a negative selection step. After selection of technology type and technical replicates the whole sample hierarchy can be downloaded in a TSV including metadata and registered to the database.

**Figure 4:**
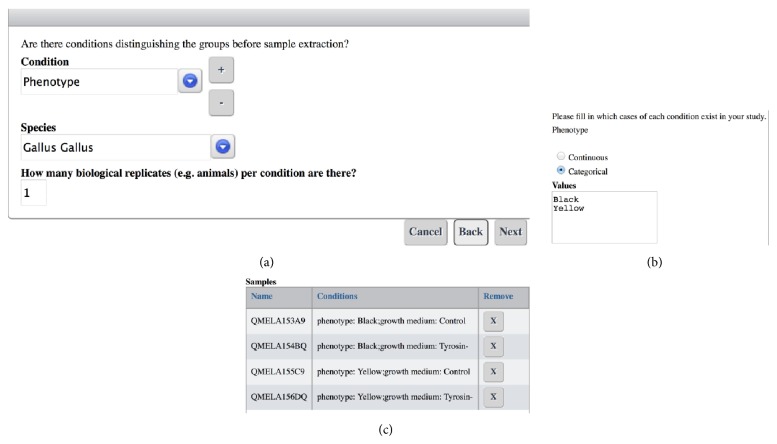
(a) Creation of biological entities using our portlet. An arbitrary number of experimental conditions can be added and the number of replicates can be chosen. (b) Dynamically generated input fields ask the user to specify the condition chosen in the last step. For continuous variables a selection of SI units is offered. (c) Users have the possibility to delete samples from the design after every step in the experimental design in the case the design is not symmetric.

We implemented a Java-based portlet with a wizard-like user front-end to enable rapid experimental design in high-throughput biology [19]. The wizard guides the user through the necessary steps (see Figure 3). Single steps provide basic information (Figure 4) and are designed not to overburden users with too much information, which can result in a long training period when using more complex software. Every multiplying step in the factorial design is followed by the possibility to deselect samples that are not part of the experimental design. These samples and their metadata are then not propagated to the next steps, making it possible to create large-scale, clearly represented experiments containing hundreds of samples in a matter of minutes.

For storing and managing data and metadata we use the open Biology Information System (openBIS) [20]. openBIS offers a structured data model for projects, experiments, samples, and datasets including the ability to apply user permission rules to collect and share experimental data in a secure way. Experiment, sample, and dataset types and their metadata are customizable and this information can be viewed and edited using either a built-in web interface or the provided API. openBIS uses a PostgreSQL database to store metadata and the structure of projects.

Our portlet provides functionality to preregister experiments and their sample instances in openBIS. Users are given the option to edit metadata at a more detailed level by downloading a* tab separated values* (TSV) file of the experiment containing one sample per row. This file contains connections between the samples and common metadata properties of the openBIS model that can be filled in by the user, including lab internal identifiers or a more specific description of the tissue extracts. Custom properties can be added in a special column. This functionality allows for project-specific sample annotation by the individual researchers and thereby allows convenient naming of samples and collection of metadata. Metadata properties are presented as pairs of labels and values and there is no limit for such pairs. Spreadsheets are the most widely used data exchange formats within laboratories and for the majority of researchers they are the most convenient way to adjust the metadata requirements, which was also taken into consideration in the development of the MAGE-TAB format. By uploading the sample TSV to the portlet, all properties can then be parsed to the predefined fields and to our XML schema and experiments and metadata can be registered to openBIS.

Additionally, sample extracts and test samples can be related to older entities or extracts already stored in the database or existing experiments copied, for example, for collecting different biological data from the same setup. In these cases samples can inherit connections to and properties of existing samples, making the design of follow-up experiments even easier.

**Figure 5:**
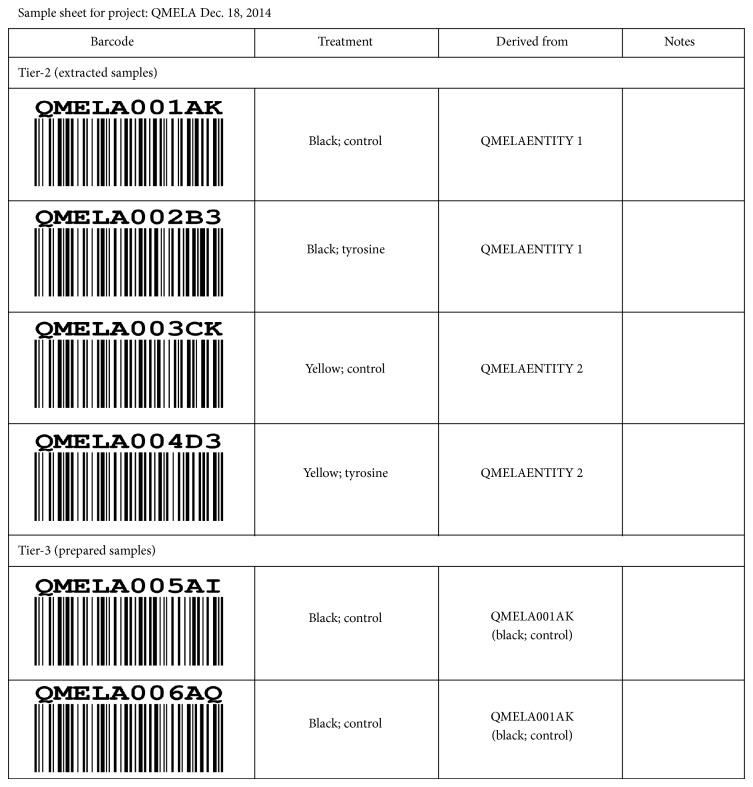
Excerpt of a sample sheet for the same experiment showing sample extracts and samples prepared for measurement. Treatment and context (derived from column) of each sample in the design are explained and scientists can add additional information.

Applications of this way of storing metadata are versatile. Apart from the often-desired possibility to download experiment information in the spreadsheet format, we can create sample sheets including identifiers, metadata, and relationships between samples for the involved labs (see Table 1 and Figure 5). Additionally, metadata can be shown in other portlets and help with visualization of the data that it belongs to or even be used to start workflows that analyze the data. For example, one of the most frequently used data processing tools in computational proteomics, MaxQuant, uses an experimental design file to assign raw data files to the experiment to which they belong [21]. This file also contains metadata details of fraction numbers and allows MaxQuant to analyze multiple files together, while still retaining the individual ratio values for each sample. Since experiment information and sample hierarchy are an integral part of our design and we can store additional metadata like fraction numbers, the wizard enables a fully automated and straightforward creation of the MaxQuant experimental design schema. With MaxQuant being only one example, we anticipate that our portlet will be widely applicable in bioinformatics pipelines where decisions of the analysis are based on the corresponding metadata.

The complete source code of our Java implementation can be downloaded from https://github.com/qbicsoftware/qwizard.

## 4. Conclusions

Advances in the creation of biological data have made it necessary to store, analyze, and describe data in new and effective ways. We suggest a factor-based approach to help scientists with the design of experiments for high-throughput biomedical data, enabling the intuitive and fast creation of large experiments before the biological data is measured. This assures that incoming data is accounted for in the design, making experiments more robust, significant, and reproducible.

The possibility to collect a multitude of metadata is important for both analysis of an experiment and future experiments or even large-scale data mining. Existing approaches like MAGE-TAB for the MIAME standard successfully make use of spreadsheet-based formats but are often limited to a single technology or field. With the rise of both multiomics integration projects and facilities that provide analysis for a multitude of technologies, a unifying approach is needed.

XML formats are easy to parse, are used in many applications, and can be validated on the fly using XML schemas. These properties enable the reusability of metadata in many different applications like visualization portlets or the creation of sample sheets. This is also achieved by the possibility to derive new experiments from samples already known to the system.

Connection to a data management system allows for context-specific input fields and instant feedback if erroneous inputs are performed. Since our implementation is web-based, there is no need to install new software, which can be problematic when different operating systems are in use or scientists do not have sufficient rights to install software. This also allows for easy access of different involved labs which can download sample sheets with identifiers and metainformation to help carry out experiments faster and with fewer errors.

The generic implementation and the flexible back-end, as served by openBIS, allow easy customizing and adapting of both the model and the portlet to better suit scientists needs.

## Figures and Tables

**Table 1:** TSV created by the experimental design portlet (see Figure 4; excerpt) showing different metadata relevant to the experiment. Empty columns can be filled or additional columns added by the user before reupload (e.g., external identifiers) or the existing information edited.

Identifier	Sample type	Parent	Q_Primary_Tissue	Q_NCBI_Organism	Q_SAMPLE_TYPE	Q_EXTERNALDB_ID	XML_FACTORS
QMELAENTITY-1	Q_BIOLOGICAL_ENTITY			9031			Phenotype: black
QMELAENTITY-2	Q_BIOLOGICAL_ENTITY			9031			Phenotype: yellow
QMELA001AK	Q_BIOLOGICAL_SAMPLE	QMELAENTITY-1	Skin				Phenotype: black; growth medium: control
QMELA002B3	Q_BIOLOGICAL_SAMPLE	QMELAENTITY-1	Skin				Phenotype: black; growth medium: tyrosin-
QMELA003CK	Q_BIOLOGICAL_SAMPLE	QMELAENTITY-2	Skin				Phenotype: yellow; growth medium: control
QMELA004D3	Q_BIOLOGICAL_SAMPLE	QMELAENTITY-2	Skin				Phenotype: yellow; growth medium: tyrosin-
QMELA005AI	Q_TEST_SAMPLE	QMELA001AK			Smallmolecules		Phenotype: black; growth medium: control
QMELA006AQ	Q_TEST_SAMPLE	QMELA001AK			Smallmolecules		Phenotype: black; growth medium: control
QMELA007B9	Q_TEST_SAMPLE	QMELA002B3			Smallmolecules		Phenotype: black; growth medium: tyrosin-
QMELA008BH	Q_TEST_SAMPLE	QMELA002B3			Smallmolecules		Phenotype: black; growth medium: tyrosin-
QMELA009C0	Q_TEST_SAMPLE	QMELA003CK			Smallmolecules		Phenotype: yellow; growth medium: control
QMELA010C3	Q_TEST_SAMPLE	QMELA003CK			Smallmolecules		Phenotype: yellow; growth medium: control
QMELA011DK	Q_TEST_SAMPLE	QMELA004D3			Smallmolecules		Phenotype: yellow; growth medium: tyrosin-
QMELA012DS	Q_TEST_SAMPLE	QMELA004D3			Smallmolecules		Phenotype: yellow; growth medium: tyrosin-
